# Retrievability of Odontopaste and Metapex With 17% Ethylenediaminetetraacetic Acid and 10% Maleic Acid From Root Canals: An Invitro Study

**DOI:** 10.7759/cureus.21508

**Published:** 2022-01-23

**Authors:** Sneha Pallepagu, Swathi Aravelli, Bhaskar Bamini, Neethu Nanda B, Narender Reddy, Ankita Reddy Amaravai

**Affiliations:** 1 Department of Conservative Dentistry and Endodontics, Malla Reddy Dental College for Women, Hyderabad, IND; 2 Department of Conservative Dentistry and Endodontics, Government Dental College and Hospital, Hyderabad, IND; 3 Department of Conservative Dentistry and Endodontics, SVS Institute of Dental Sciences, Mahabubnagar, IND; 4 Department of Conservative Dentistry and Endodontics, SVS institute of Dental Sciences, Hyderabad, IND

**Keywords:** stereomicroscope, retrieval, maleic acid, intracanal medicament, edta, calcium hydroxide

## Abstract

Aim

To evaluate the efficacy of 10% maleic acid in comparison with 17% ethylenediaminetetraacetic acid (EDTA) in the removal of intracanal medicaments from the root canal system.

Materials and methods

Forty-eight extracted single-rooted mandibular premolars were decoronated to standardize the length of 14 mm. Chemomechanical preparation was done using the crown-down technique with Protaper files (Dentsply‑Maillefer, Ballaigues, Switzerland) till F4, followed by irrigation with 2 ml of 5.25% sodium hypochlorite (NaOCl) after each instrument, and 5 ml of 17% EDTA was used as the final irrigating agent.

Metapex (Meta Dental Corp. Ltd., Elmhurst, NY, USA) and Odontopaste (Australian Dental Manufacturing, Kenmore Hills, Qld, Australia) were the two intracanal medicaments that were used in this study.

Total samples were divided into two groups based on the intracanal medicament that was placed in the canal. In group 1, Metapex was injected into the root canal until the material extruded through the apex. In group 2, Odontopaste was placed into the canal until the material extruded through the root apex. Cleaning off the excess medicament was done with a moist cotton pellet. After temporary sealing with a cotton pellet and Cavit, all the samples were stored at 37 ºC and 100% relative humidity for a period of seven days. The teeth in each group were further randomly divided into three subgroups on the basis of the irrigant used for retrieval of medicament. In groups 1A and 2A, 1ml of 17% EDTA was used; in groups 1B and 2B, 1ml of 10% maleic acid was used; in groups 1C and 2C, 1ml of 0.9% saline was used. Sonic agitation for 1 minute, followed by a final rinse of 1 ml distilled water, was used in all the groups.

After the intracanal medicament was removed from the canal, the roots were longitudinally sectioned using a diamond disk (Bego, Berman, Germany). The residual medicament on each section was evaluated under a stereomicroscope (×30; Medilux, MDL-DS4-BI, Biosystems, Curitiba, PR, Brazil). The data were analyzed using SPSS version 23.0 software (IBM Corp., Armonk, NY). Kruskal-Wallis ANOVA and Mann-Whitney U test (post hoc) were applied for intergroup comparisons. A Wilcoxon signed-rank test was applied for intragroup comparisons.

Results

Both the chelators, 17% EDTA and 10% maleic acid, removed the Odontopaste significantly better than Metapex. However, 17% EDTA was more effective in the removal of Odontopaste. 10% Maleic acid showed better results in the removal of Metapex than 17% EDTA.

Conclusion

None of the chelating agents was able to totally retrieve the intracanal medicaments. When compared to Metapex, Odontopaste showed significantly better retrievability from the root canal with both 17% EDTA and 10% Maleic acid, whereas the retrievability of Metapex was significantly better with 10% Maleic acid in comparison to 17% EDTA.

## Introduction

Root canal treatment is specifically directed towards the control and prevention of pulpal and periradicular diseases. The prognosis of root canal treatment depends on the reduction or eradication of microorganisms, as they are relevant in the progression of these lesions [1].

The number of microorganisms in the infected root canal was significantly reduced by chemomechanical preparation. However, intracanal dressing enhances the elimination of microorganisms from the infected root canals by preventing the recontamination and proliferation of residual strains [2].

Presently, calcium hydroxide is the most popular intracanal medicament. It is widely used as an interappointment root canal dressing due to its well-documented antimicrobial activity against most endodontic microorganisms. Most of the bacteria are unable to survive the extremely alkaline environment provided by calcium hydroxide [3].

Prior to obturation, the intracanal medicament is removed from the root canal, as the retained medicament may obstruct the penetration of sealer into the dentinal tubules, thereby increasing the risk of apical microleakage [4,5]. Calcium hydroxide is the most widely used intracanal medicament due to its well-documented antibacterial activity and is available in various combinations. Metapex is the most commonly used medicament among these combinations and is silicone oil-based calcium hydroxide containing 38% iodoform [6].

Odontopaste (Australian Dental Manufacturing, Kenmore Hills, Qld, Australia) is a zinc oxide-based dressing that consists of 5% Clindamycin hydrochloride and 1% triamcinolone acetonide. In addition to the benefits of zinc oxide paste, the antibiotic provides bacteriostatic activity, thereby preventing bacterial repopulation within the canal. Odontopaste also helps in reducing inflammation due to the presence of the steroid component triamcinolone acetonide [7].

The intracanal medicament may remain in the canal irregularities as they are inaccessible to conventional irrigation procedures. Several techniques have been recommended, such as sonics and ultrasonics, in order to enhance the irrigation phase and thereby improve the efficacy of intracanal medicaments. The endoactivator system (Dentsply Tulsa Dental Specialties, Tulsa, OK, USA) is a sonically driven canal irrigation system and produces rapid intracanal fluid agitation upon its activation. Compared to traditional needle irrigation, this endoactivator system has been shown to provide better irrigation of the lateral canals at 4.5 mm and 2 mm of working length [8].

This study aims to evaluate the retrievability of Odontopaste and Metapex with 17% ethylenediaminetetraacetic acid (EDTA) and 10% maleic acid from root canals using a stereomicroscope.

## Materials and methods

Forty-eight extracted single-rooted mandibular premolars free of cracks, fractures, or any other defect were included in this study. Decoronation of the teeth was done to standardize the length of 14 mm. Chemomechanical preparation was done using the crown-down technique with protaper files (Densply‑Maillefer, Ballaigues, Switzerland) till F4, followed by irrigation with 2 ml of 5.25% sodium hypochlorite (NaOCl) after each instrument, and 5 ml of 17% EDTA was used as the final irrigating agent. Drying of the canals was done with paper points (Densply-Maillefer, Ballaigues, Switzerland).

The two intracanal medicaments used in this study were Metapex (Meta Dental Corp. Ltd., Elmhurst, NY, USA) and Odontopaste (Australian Dental Manufacturing, Kenmore Hills, Qld, Australia).

The total samples were divided into two groups based on the intracanal medicament that was placed in the canal. In group 1 (n=24), Metapex was injected into the root canal until the material extruded through the apex. In group 2 (n=24), Odontopaste was placed into the canal with lentulospiral until the material extruded through the root apex. Cleaning off the excess medicament was done with a moist cotton pellet. After temporary sealing with a cotton pellet and Cavit (ESPE Dental, Seefeld, Germany), all the samples were stored at 37 ºC and 100% relative humidity for a period of seven days.

In each group, the teeth were further randomly divided into three subgroups on the basis of irrigant used for retrieval of the medicament.

Group 1A (n = 8): Metapex removed with 1 ml of 17% EDTA + sonic agitation for 1 min + final rinse with 1 ml of distilled water.

Group 1B (n = 8): Metapex removed with 1 ml of 10% Maleic acid + sonic agitation for 1 min + final rinse with 1 ml of distilled water.

Group 1C (n = 8): Metapex removed with 1 ml of 0.9% saline + sonic agitation for 1 min + final rinse with1 ml of distilled water.

Group 2A (n = 8): Odontopaste removed with 1 ml of 17% EDTA + sonic agitation for 1 min + final rinse with 1 ml of distilled water.

Group 2B (n = 8): Odontopaste removed with 1 ml of 10% Maleic acid + sonic agitation for 1 min + final rinse with 1 ml of distilled water.

Group 2C (n = 8) the canal: Odontopaste removed with 1 ml of 0.9% saline + sonic agitation for 1 min + final rinse with 1 ml of distilled water.

After the intracanal medicament was removed from the canal, on the buccal and lingual parts of the root, two slots were prepared using a diamond disk (Bego, Berman, Germany), followed by subjecting the roots to longitudinal sectioning. The residual medicament on each section was evaluated under a stereomicroscope (×30; Medilux, MDL-DS4-BI, Biosystems, Curitiba, PR, Brazil).

The remaining intracanal medicament on the canal wall was assessed by using a scoring system adopted by Lambrianidis et al. [9]. (i) Score 1 - no visible remains of intracanal medicament, equal to the negative control group. (ii) Score 2 - remnants of intracanal medicament dispersed in small quantities on the root canal walls. (iii) Score 3 - masses of intracanal medicament in different areas, moderate waste. (iv) Score 4 - dense masses of intracanal medicament across the root canal wall.

The data were analyzed using SPSS version 23.0 software (IBM Corp., Armonk. NY). The data follow "a non-normal" distribution. Hence, non-parametric tests of significance were applied. Kruskal-Wallis ANOVA and Mann-Whitney U test (post hoc) were applied for intergroup comparisons. A Wilcoxon signed-rank test was applied for intragroup comparisons. A p-value of 0.05 was considered statistically significant.

## Results

**Figure 1:**
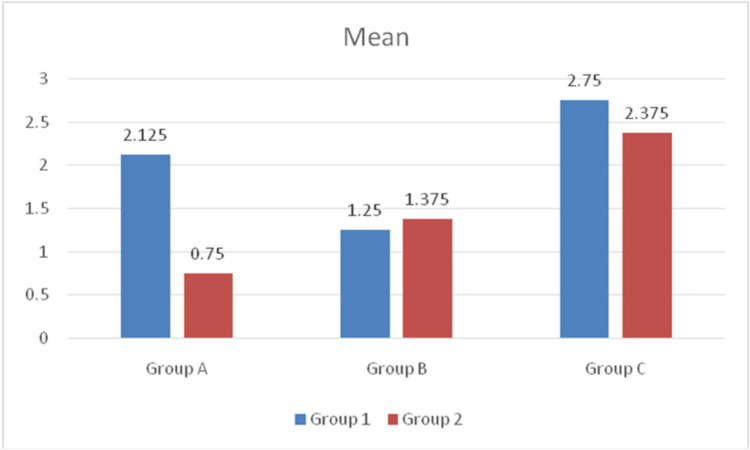
Comparison between groups A significant difference was observed between the 2A-2C pair and the 2B-2C pair. Intragroup comparisons showed significant differences between 1A and 2A groups.

In group 1, the mean of 1C (2.75 ± 0.46) is high, followed by 1A (2.12 ± 0.64) and the least for 1B (1.25 ± 0.70). Significant differences were observed between the 1A-1B pair and the 1B-1C pair. In group 2, the mean of 2C is high (2.37 ± 0.51) followed by 2B (1.37 ± 53) and the least for 2A (0.74 ± 0.46). A significant difference was observed between the 2A-2C pair and the 2B-2C pair (Table 1 and Figure 1).

**Table 1:** Intergroup comparison: Kruskal-Wallis ANOVA and Mann-Whitney U test (post hoc) N: number of samples; std. deviation: standard deviation; p-value: probability value for level of significance; HS: highly significant at p<0.01.

		N	Mean	Std. deviation	Minimum	Maximum	Chi-square	p-value	Significant pairs
Group 1	Group 1A	8	2.1250	0.64087	1.00	3.00	12.66	0.002 HS	(1A, 1B), (1B, 1C)
	Group 1B	8	1.2500	0.70711	0.00	2.00			
	Group 1C	8	2.7500	0.46291	2.00	3.00			
Group 2	Group 2A	8	0.7500	0.46291	0.00	1.00	16.31	0.000 HS	(2A, 2C), (2B, 2C)
	Group 2B	8	1.3750	0.51755	1.00	2.00			
	Group 2C	8	2.3750	0.51755	2.00	3.00			

Intragroup comparisons showed significant differences between the 1A and 2A groups (p=0.016). The mean of 1A is greater than 2A (Table 2 and Figure 1).

**Table 2:** Intragroup comparison: Wilcoxon signed-rank test Z-value: standard score; S: significant at p<0.05.

		Mean	Std. deviation	Z-value	p-value
Pair 1	1A	2.1250	0.64087	−2.41	0.016 S
	2A	0.7500	0.46291		
Pair 2	1B	1.2500	0.70711	−0.57	0.56
	2B	1.3750	0.51755		
Pair 3	1C	2.7500	0.46291	−1.73	0.08
	2C	2.3750	0.51755		

Both the chelators, 17% EDTA and 10% maleic acid, removed the Odontopaste significantly better than Metapex. However, 17% EDTA was more effective in the removal of Odontopaste. Both 17% EDTA and 10% Maleic acid were equally good in the removal of Odontopaste, but for Metapex, 10% Maleic acid was better than 17% EDTA. Saline irrigation was the least effective in the removal of both the intracanal medicaments.

## Discussion

Microorganisms in the infected root canal are decreased by thorough instrumentation along with irrigation [10]. However, intracanal medicament enhances the eradication of microorganisms from the canal irregularities, thereby preventing the proliferation of residual bacterial strains.

The most widely used intracanal medicament to date is calcium hydroxide due to its well-documented antimicrobial activity against most of the pathogenic strains present in root canal infections. Most in vitro studies have reported that remnants of calcium hydroxide can markedly increase apical leakage by obstructing the penetration of sealer into dentinal tubules. It also makes the zinc oxide eugenol sealer brittle and granular by potentially interacting with it [11,12].

Copious needle irrigation with sodium hypochlorite along with hand instrumentation was used in order to remove intracanal medicament from the root canal, followed by a final rinse with EDTA. However, these techniques are considered to be inefficient to completely eliminate intracanal medicament. In addition to removing the smear layer, EDTA also chelates calcium from calcium hydroxide intracanal medicament. Studies have shown that EDTA is more erosive to dentin when compared to other chelating agents such as 10% maleic acid [13].

None of the chelating agents tested in this study were able to remove intracanal medicament completely. Some of the in vitro studies have reported that the type of vehicle present in the medicament can affect the removal efficacy of the chelating agent [14]. It was shown that 17% EDTA and 10% maleic acid solution removed Odontopaste more effectively than Metapex.

Metapex contains an oily vehicle, i.e., silicon oil, which might have restricted its dissolution and removal from the root canal by the tested chelators. The 10% maleic acid solution performed better in comparison to the 17% EDTA solution in the removal of Metapex. This could probably be because of the ability of maleic acid to penetrate the silicone oil in comparison to EDTA and chelate the calcium ions. However, both 17% EDTA and 10% maleic acid solutions were effective in the removal of Odontopaste because of the aqueous-based vehicle (polyethylene glycol) present in it [13].

In the Metapex group, where silicone oil was used as a vehicle, 10% maleic acid showed better retrieval capability of medicament than 17% EDTA. Maleic acid has less surface tension than EDTA. In this study, during initial root canal preparation, 17% EDTA and 5.25% sodium hypochlorite were used in combination for irrigation so as to remove the smear layer [15]. It is known that sodium hypochlorite and EDTA have similar surface tension levels, which are important for wetting capability and effectiveness in the removal of the smear layer. This might have enhanced the effect of maleic acid. The acidic nature and low surface tension of maleic acid might have also resulted in better penetration into the dentinal tubules.

Limitations of the study

After retrieval of the medicament from the canal, only the surface area of the canal wall covered with intracanal medicament was analyzed in this study, not the volume. In addition, loss of medicament may occur during sectioning of teeth, which may influence the results of the study. Retrieval of intracanal medicament by chelating agent may vary at the apical third and middle third of the root canal, which was not considered in the present study.

## Conclusions

Within the limitations of the study, none of the chelating agents was able to totally retrieve the intracanal medicaments. Among the medicaments tested, Odontopaste showed better retrievability than Metapex with both 17% EDTA and 10% Maleic acid. The vehicle used in the intracanal medicament might have influenced its retrievability.
